# FIGO good practice recommendations on delayed umbilical cord clamping

**DOI:** 10.1002/ijgo.13841

**Published:** 2021-09-14

**Authors:** Ana Bianchi, Bo Jacobsson, Ben W. Mol, the FIGO Working Group for Preterm Birth, Bo Jacobsson, Joe Leigh Simpson, Jane Norman, William Grobman, Ana Bianchi, Stephen Munjanja, Catalina María Valencia González, Ben W. Mol, Andrew Shennan

**Affiliations:** ^1^ Perinatal Department Pereira Rossell Hospital Public Health and University Hospital Montevideo Uruguay; ^2^ Department of Obstetrics and Gynecology Institute of Clinical Science Sahlgrenska Academy University of Gothenburg Gothenburg Sweden; ^3^ Department of Obstetrics and Gynecology Sahlgrenska University Hospital Gothenburg Sweden; ^4^ Department of Genetics and Bioinformatics Domain of Health Data and Digitalization Institute of Public Health Oslo Norway; ^5^ Department of Obstetrics and Gynecology Monash University Clayton Australia; ^6^ Aberdeen Centre for Women's Health Research Institute of Applied Health Sciences School of Medicine Medical Sciences and Nutrition University of Aberdeen Aberdeen UK

**Keywords:** delayed cord clamping, neonatal outcomes, preterm delivery, term delivery

## Abstract

Delayed cord clamping in the first minute in preterm infants born before 34 weeks of gestation improves neonatal hematologic measures and may reduce mortality without increasing any other morbidity. In term‐born babies, it also seems to improve both the short‐ and long‐term outcomes and shows favorable scores in fine motor and social domains. However, there is insufficient evidence to show what duration of delay is best. The current evidence supports not clamping the cord before 30 seconds for preterm births. Future trials could compare different lengths of delay. Until then, a period of 30 seconds to 3 minutes seems justified for term‐born babies.

## Synopsis

Delayed cord clamping in the first minute in preterm infants born before 34 weeks of gestation improves neonatal hematologic measures and may reduce mortality without increasing any other morbidity.

## 1 INTRODUCTION

Since active management of the third stage of labor was introduced, early clamping of the umbilical cord has spread across the world. However, during the last decade, clinical insights have questioned this policy. This gained support through extensive clinical trials showing that late cord clamping results in better neonatal outcomes in preterm and term‐born babies.^1^, ^2^ Delayed cord clamping allows blood flow between the placenta and baby to continue during the third stage of labor, leading to a more stable neonatal hemodynamic transition.^3^ In addition, as part of the neonate’s physiological transition naturally occurring during the third stage of labor there is a progressive increase in heart rate, which is now considered the most reliable indicator of normalcy. In this sense, delayed cord clamping provides a safe time lapse to avoid rushing to perform unnecessary interventions.^4^

A recent study by Katheria et al.^5^ found that in preterm infants born before 32 weeks of gestation more than 90% of infants established respirations with colorimetric carbon dioxide change within the first minute after birth when they received stimulation. This is important for cardiovascular stability to occur; the newborn must be breathing, therefore during delayed cord clamping attempts should be made to get them to breathe spontaneously. This, as a minimum, should be conducted with stimulation and should allow a safe time to assess the slow initiation of spontaneous breathing and to provide minimally invasive medical support, if needed, avoiding unnecessary and potentially harmful interventions.

The shorter the gestational age, the more significant the delay in initiating effective breathing owing to an immature respiratory drive, poor muscle strength, and surfactant deficiency. The initial functional residual lung capacity provides an insignificant amount of pulmonary exchange during the first breaths. Safety of respiration may depend transiently on the placental gas exchange that makes a substantial contribution to the infant’s oxygen needs during these seconds and minutes of transition.

Caring for the preterm infant with an intact umbilical cord and in the “maternal space” allows a safe time for assessing the slow initiation of spontaneous breathing.

## 2 CLINICAL SCENARIOS

### 2.1 Delayed cord clamping at preterm birth

Systematic reviews provide moderate‐quality evidence that delayed cord clamping in the first minute in preterm infants born before 34 weeks of gestation improves neonatal hematologic measures and may reduce mortality without increasing any other morbidity.^6^

Delayed clamping reduced hospital mortality (risk ratio [RR] 0.68; 95% CI 0.52–0.90; risk difference –0.03; 95% CI –0.05 to –0.01; *P* = 0.005; number needed to benefit 33; 95% CI 20–100).^7^ In three trials including 996 infants at or before 28 weeks of gestation, delayed cord clamping reduced hospital mortality (RR 0.70; 95% CI 0.51–0.95; risk difference –0.05; 95% CI –0.09 to –0.01; *P* = 0.02; number needed to benefit 20; 95% CI 11–100).^7^ Delayed clamping reduced the incidence of a low Apgar score at 1 minute, but not at 5 minutes, and did not reduce the incidence of intubation for resuscitation, admission temperature, mechanical ventilation, intraventricular hemorrhage, brain injury, chronic lung disease, patent ductus arteriosus, necrotizing enterocolitis, late‐onset sepsis, or retinopathy of prematurity.^7^

### 2.2 Delayed cord clamping at term birth

Although not the focus of these recommendations, there is also evidence that expectant management in full‐term babies is beneficial in the third stage of labor in the short and long term.^8^ In the short term, delayed cord clamping increases early hemoglobin concentrations and iron stores in infants.^8^ In the long term, delayed cord clamping is likely to be beneficial as long as access to treatment for jaundice requiring phototherapy is available.

A randomized trial of full‐term infants from low‐risk pregnancies in a high‐income country assessed neurodevelopment at 4 years and compared delayed versus early cord clamping. Favorable scores for delayed cord clamping were found in the fine motor and social domains at 4 years of age, especially in boys.^2^ Prevention of iron deficiency in infancy may promote neurodevelopment. Delayed umbilical cord clamping prevents iron deficiency at 4–6 months of age, and long‐term effects have yet to be reported.^8^ Some trials have also followed the impact of cord clamping on the early developing brain at 12 months in a healthy population, concluding that infants who received delayed cord clamping had greater myelin content in brain regions involving motor, function, visual, spatial, and sensory processing.^9^

## 3 RECOMMENDATIONS

Delayed cord clamping in the first minute in preterm infants born before 34 weeks of gestation improves neonatal hematologic measures and may reduce mortality without increasing any other morbidity. In term‐born babies, it also seems to improve the short‐ and long‐term outcomes and showed favorable scores in the fine motor and social domains at 4 years of age.

However, there is insufficient evidence to show what duration of delay is best. The current evidence supports not clamping the cord before 30 seconds for preterm births. Future trials could compare different lengths of delay. Until then, at term a period of 30 seconds to 3 minutes seems justified or until the cord is collapsed and white.

For both preterm and term‐born babies, during the cord clamping delay, attempts should be made to get them to breathe spontaneously. Additional research is needed to examine the long‐term child outcome related to the timing of umbilical cord clamping and the developing brain.

## Conflicts of interest

Ana Bianchi reports no conflicts of interest. Ben W. Mol reports an investigator grant from NHMRC; consultancy for ObsEva; and research funding from Guerbet, Ferring, and Merck KGaA. Bo Jacobbson reports research grants from Swedish Research Council, Norwegian Research Council, March of Dimes, Burroughs Wellcome Fund, and the US National Institute of Health; clinical diagnostic trials on NIPT with Ariosa (completed), Natera (ongoing), Vanadis (completed), and Hologic (ongoing) with expenditures reimbursed per patient; clinical probiotic studies with product provided by FukoPharma (ongoing, no funding), and BioGaia (ongoing; also provided a research grant for the specific study); collaboration in IMPACT study where Roche, Perkin Elmer, and Thermo Fisher provided reagents to PLGF analyses; coordination of scientific conferences and meetings with commercial partners such as NNFM 2015, ESPBC 2016, and a Nordic educational meeting about NIPT and pre‐eclampsia screening. Bo Jacobbson is also Chair of the FIGO Working Group for Preterm Birth and the European Association of Perinatal Medicine special interest group on preterm delivery; steering group member of Genomic Medicine Sweden; chairs the Genomic Medicine Sweden complex diseases group; and is Swedish representative in the Nordic Society of Precision Medicine.

## Author Contributions

All authors and the FIGO Working Group for Preterm Birth drafted the concept for the paper. BWM and AB wrote the first version and BJ revised various versions of the manuscript. All authors and working group members commented on the manuscript and approved the final version.

## MEMBERS Of THE FIGO WORKING GROUP FOR PRETERM BIRTH, 2018–2021

Bo Jacobsson (Chair), Joe Leigh Simpson, Jane Norman, William Grobman, Ana Bianchi, Stephen Munjanja, Catalina María Valencia González, Ben W. Mol, Andrew Shennan.
